# Emerging role of non-coding RNAs in the regulation of Sonic Hedgehog signaling pathway

**DOI:** 10.1186/s12935-022-02702-y

**Published:** 2022-09-13

**Authors:** Soudeh Ghafouri-Fard, Tayyebeh Khoshbakht, Bashdar Mahmud Hussen, Mohammad Taheri, Majid Samsami

**Affiliations:** 1 Department of Medical Genetics, School of Medicine, Shahid Beheshti University of Medical Sciences, Tehran, Iran; 2 Phytochemistry Research Center, Shahid Beheshti University of Medical Sciences, Tehran, Iran; 3 Department of Pharmacognosy, College of Pharmacy, Hawler Medical University, Kurdistan Region Erbil, Iraq; 4 Center of Research and Strategic Studies, Lebanese French University, Erbil, Kurdistan Region, Iraq; 5 Institute of Human Genetics, Jena University Hospital, Jena, Germany; 6 Urology and Nephrology Research Center, Shahid Beheshti University of Medical Sciences, Tehran, Iran; 7 Cancer Research Center, Shahid Beheshti University of Medical Sciences, Tehran, Iran

**Keywords:** Shh signaling, Sonic Hedgehog signaling, Long non-coding RNA, miRNA

## Abstract

Sonic Hedgehog (Shh) signaling cascade is one of the complex signaling pathways that control the accurately organized developmental processes in multicellular organisms. This pathway has fundamental roles in the tumor formation and induction of resistance to conventional therapies. Numerous non-coding RNAs (ncRNAs) have been found to interact with Shh pathway to induce several pathogenic processes, including malignant and non-malignant disorders. Many of the Shh-interacting ncRNAs are oncogenes whose expressions have been increased in diverse malignancies. A number of Shh-targeting miRNAs such as miR-26a, miR-1471, miR-129-5p, miR-361-3p, miR-26b-5p and miR-361-3p have been found to be down-regulated in tumor tissues. In addition to malignant conditions, Shh-interacting ncRNAs can affect tissue regeneration and development of neurodegenerative disorders. XIST, LOC101930370, lncRNA-Hh, circBCBM1, SNHG6, LINC‐PINT, TUG1 and LINC01426 are among long non-coding RNAs/circular RNAs that interact with Shh pathway. Moreover, miR-424, miR-26a, miR-1471, miR-125a, miR-210, miR-130a-5p, miR-199b, miR-155, let-7, miR-30c, miR-326, miR-26b-5p, miR-9, miR-132, miR-146a and miR-425-5p are among Shh-interacting miRNAs. The current review summarizes the interactions between ncRNAs and Shh in these contexts.

## Introduction

Sonic Hedgehog (Shh) signaling cascade is one of the complicated signaling pathways that administrate the accurately controlled developmental processes in multicellular organisms. It has an important role in the establishment of the outlines of cellular differentiation to regulate multifaceted organ formation. This pathway affects these cellular processes via a cascade that changes the equilibrium between activator and repressor types of glioma-associated oncogene (Gli) transcription factors. A number of Hedgehog (Hh) ligands as well as Patched receptors, Smoothened receptor, Suppressor of fused homolog, Kif7, PKA and cAMP participate in the transfer of signals to the Gli transcription factors. Transfer of the activator form of Gli to the nucleus and its binding with the promoters of target genes lead to the stimulation of the transcription of these genes [1]. *Hh* gene has been firstly discovered about four decades ago via genetic screen experiments in Drosophila [2].

Shh participates in the tissue regeneration processes and repair mechanism in the post-embryonic period. This pathway has a crucial role in the induction of diverse populations of neurons in the central nervous system, governing several morphogenetic processes in this system [3].

Abnormal regulation of these signals has been shown to be associated with congenital malformations, aberrant tissue regeneration, stem cell renewal and carcinogenesis [4]. Expression, cellular uptake and translocation of the Shh protein as a key Hh ligand precursor have important effects in the regulatory function of Shh signaling [5]. Two other Hedgehog homologues, namely Desert (Dhh) and Indian (Ihh) have been identified in mammals.

Functional studies have shown the importance of Shh signaling in ventral cell type induction. Moreover, disruption of this pathway and recessive mutations have led to cyclopia and severe holoprosencephaly in mice, respectively [6]. In human, heterozygote mutations in Shh have been associated with different clinical features of holoprosencephaly [7].

Shh signaling is also implicated in the regulation of function of normal adult stem cells as well as cancer stem cells [8]. Dysregulation of the Hh signaling pathway has been linked with developmental abnormalities including Gorlin syndrome [9] and cancer [10, 11]. Abnormal activity of this pathway is also involved in the tumor formation and induction of resistance to radio/chemotherapy [12]. Thus, efforts have been made to find novel Shh signaling inhibitors to combat these features [12].

More recently, numerous non-coding RNAs (ncRNAs) have been found to interact with Shh pathway to induce several pathogenic processes, including malignant and non-malignant disorders. The current review summarizes the interactions between ncRNAs and Shh in these contexts. Long non-coding RNAs (lncRNAs), microRNAs (miRNAs) and circular RNAs (circRNAs) are three main regulatory ncRNAs which are discussed in this context. LncRNAs are transcripts with sizes more than 200 nt that regulate expression of genes at different levels. They can regulate chromatin function, influence the assemblage and functions of membraneless nuclear bodies, control the stability and expression of cytoplasmic mRNAs and interfere with signaling pathways [13]. miRNAs have about 22 nt and mainly affect gene expression at post transcriptional level [14]. Finally, circRNAs are made by either typical spliceosome-mediated or lariat-type splicing. They can regulate expression of genes through different mechanisms [15].

For the purpose of preparation of the current review, we searched Google Scholar and PubMed databases with the key words “Shh signaling” OR “Sonic Hedgehog” AND “lncRNA” OR “miRNA” OR “circRNA”. Then, we assessed the abstract of retrieved articles to validate their relevance with the topic. We included studies that assessed function of ncRNAs in cell lines, animal model or clinical samples. A total of 50 studies were included in this review article.

## Cell line studies

### Non-malignant disorders

The importance of interactions between ncRNAs and Shh pathway has been assessed in different cell lines. This type of interaction has been found to be implicated in the pathophysiology of alopecia. This speculation is based on the results of three-dimensional culture of dermal papilla cells, a group of cells that induce regeneration of hair follicles. Experiments in this type of culture have verified up-regulation of XIST lncRNA and Shh and down-regulation of miR-424. Mechanistically, XIST has been found to sponge miR-424 to increase Shh expression. XIST silencing has led to inhibition of activity of dermal papilla cells, suppression of their proliferation and reduction of ALP activity. In fact, XIST silencing has inhibited Shh mediated hedgehog signals through affecting expression of miR-424 [16].

### Human development

Shh-interacting ncRNAs are also involved in the developmental processes. For instance, serum response factor (SRF) controls lineage specification of embryonic stem cell progenitor cells through miR-210-mediated gene silencing. Up-regulation of miR-210 in murine embryonic stem cells-originated embryoid bodies has suppressed cell growth and blocked expression of cardiac progenitor proteins Nkx2.5 and Gata4 and terminal differentiated contractile markers Mlc2v and βMHC. On the other hand, miR-210 silencing has led to activation of cardiac progenitor gene. The effect of miR-210 is exerted through decreasing activity of Shh signaling, which nurtures the cardiac progenitor program. Mechanistically, miR-210 silences Shh activity through targeting 3' UTR of Shh transcript [17]. Activation of Shh/Gli1 signaling pathway through miR-130a-5p/Foxa2 axis has been shown to affect development of fetal lung, thus being involved in the pathogeensis of congenital diaphragmatic hernia [18]. miR-199 is another miRNA that participate in craniofacial development through modulation of Shh pathway [19].

### Cancer

**Fig. 1:**
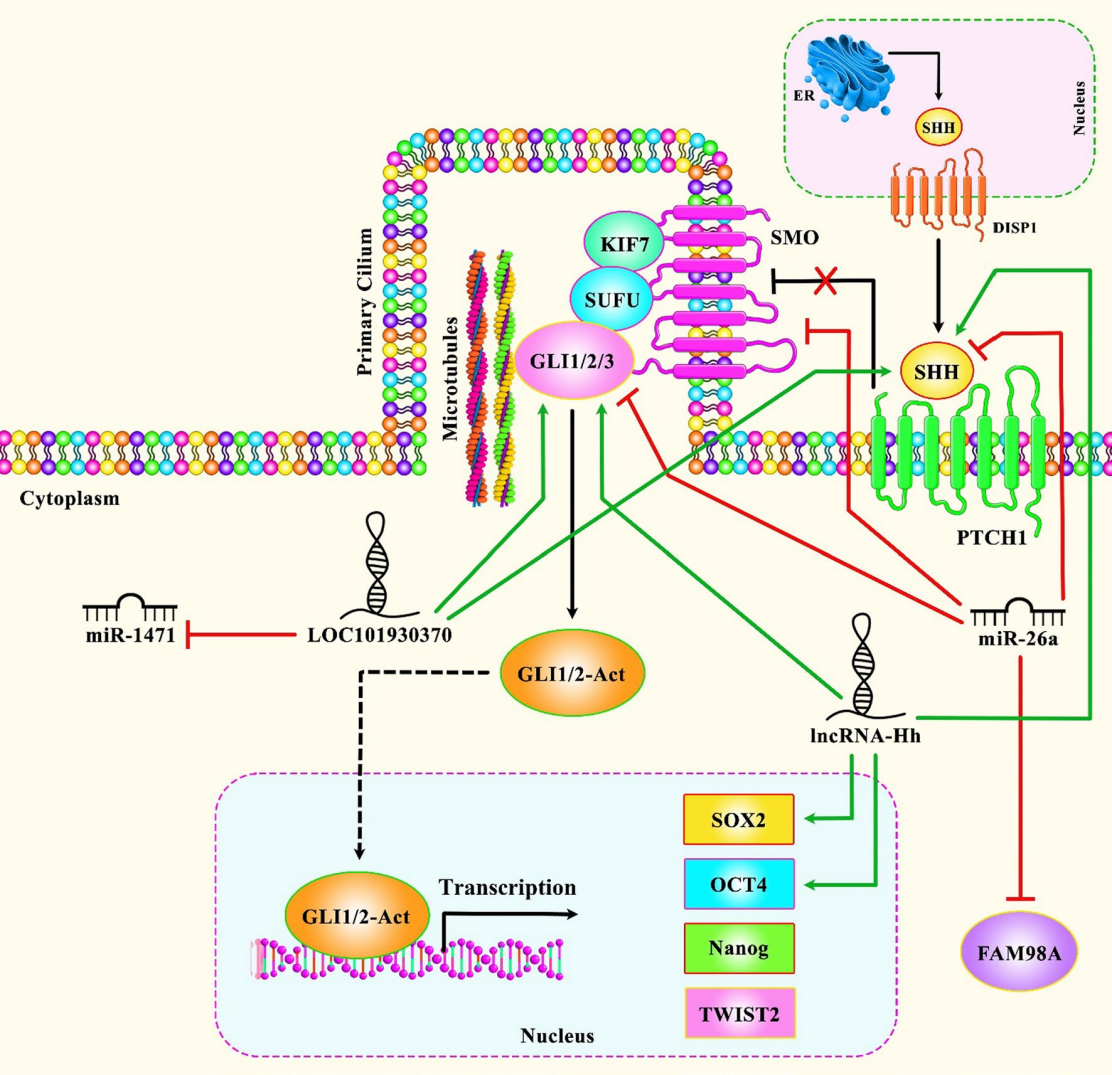
A schematic diagram of the role of several ncRNAs in triggering the Sonic Hedgehog signaling cascade in Breast Cancer. Mounting researches suggest that dysregulation of sonic hedgehog signaling pathway can play a key role in tumorigenesis in breast cancer cells. As an illustration, the recent study has detected that overexpression of lncRNA-Hh can activate Shh-GLI1 signaling and promote the expression levels of SOX2 and OCT4, thereby enhancing cancer stem cells generation in Twist-positive breast cancer cells [22]. Further experiment has validated that lncRNA LOC101930370 can significantly elevate SHH and Gli-1 expression via sponging miR-1471, therefore promoting cell proliferation and metastasis in breast cancer cells by modulating the hedgehog cascade [21]. Moreover, another research has pointed out that miR-26a has a remarkable part in suppressing breast cancer cell proliferation and invasion via downregulating the expression levels of FAM98A, SHH, SMO and GlI1, thereby inactivating the sonic hedgehog pathway in tumor cells [20]

The interaction between ncRNAs and Shh signaling pathway has also been assessed in cancer cell lines. For instance, the tumor suppressor miRNA miR-26a has been found to be down-regulated in breast cancer cell lines. Up-regulation of miR-26a has led to blockade of cell proliferation, clone formation capacity and metastatic aptitude of breast cancer cells, and induction of sensitivity to docetaxel. miR-26a could directly target FAM98A. Up-regulation of this miRNA has resulted in down-regulation of FAM98A, SHH, SMO and GLI1. Taken together, miR-26a suppresses breast carcinogenesis through inhibiting expression of FAM98A, and decreasing activity of Shh pathway [20]. miR-1471 is another down-regulated miRNA in breast cancer cells. This miRNA has been found to be sponged by LOC101930370. LOC101930370 silencing has suppressed progression of breast cancer, while inhibition of miR-1471 has increased aggressive and metastatic abilities of MCF-7 cells. Furthermore, expression levels of SHH and Gli-1 have been significantly decreased following LOC101930370 silencing, and increased by miR-1471 inhibition. Cumulatively, LOC101930370 has been found to increase expression of SHH through sponging miR-1471 [21]. Another study has revealed dysregulation of several lncRNAs in Twist-positive mammosphere cells in breast cancer cell lines. Notably, the Shh-GLI1-related lncRNA-Hh has been among these lncRNAs. Expression of this lncRNA is regulated by Twist. Moreover, lncRNA-Hh can directly target GAS1 to induce Hh activity, which in turn enhances expression of GLI1, and increases SOX2 and OCT4 levels to regulate maintenance of cancer stem cells. The latter is reflected in enhancement of mammosphere-formation efficiency and self-renewal ability in cell lines. Knock down of lncRNA-Hh in Twist-positive breast cancer cells has attenuated activity of Shh-GLI1 signaling and decreased levels of SOX and OCT4 [22]. CircBCBM1 is another example of ncRNAs that can promote metatstatic ability of breast cancer cells through acting as a molecular sponge for miR-125a and modulating expression of BRD4. This circRNA also up-regulate MMP9 levels through enhancing activity of Shh pathway [23]. Figure 1 shows the role of Shh-interacting ncRNAs in breast cancer.

In pancreatic cancer cell lines, miR-132 expression has been found to be up-regulated parallel with down-regulation of Shh levels. Besides, miR-132 mimics could significantly decrease expression of Shh at both transcript and protein levels, facilitating proliferation of pancreatic cancer cells, which has been accompanied by down-regulation of Cyclin-D1, cleaved Caspase-3/9, and suppression of cell apoptosis [24].

**Fig. 2:**
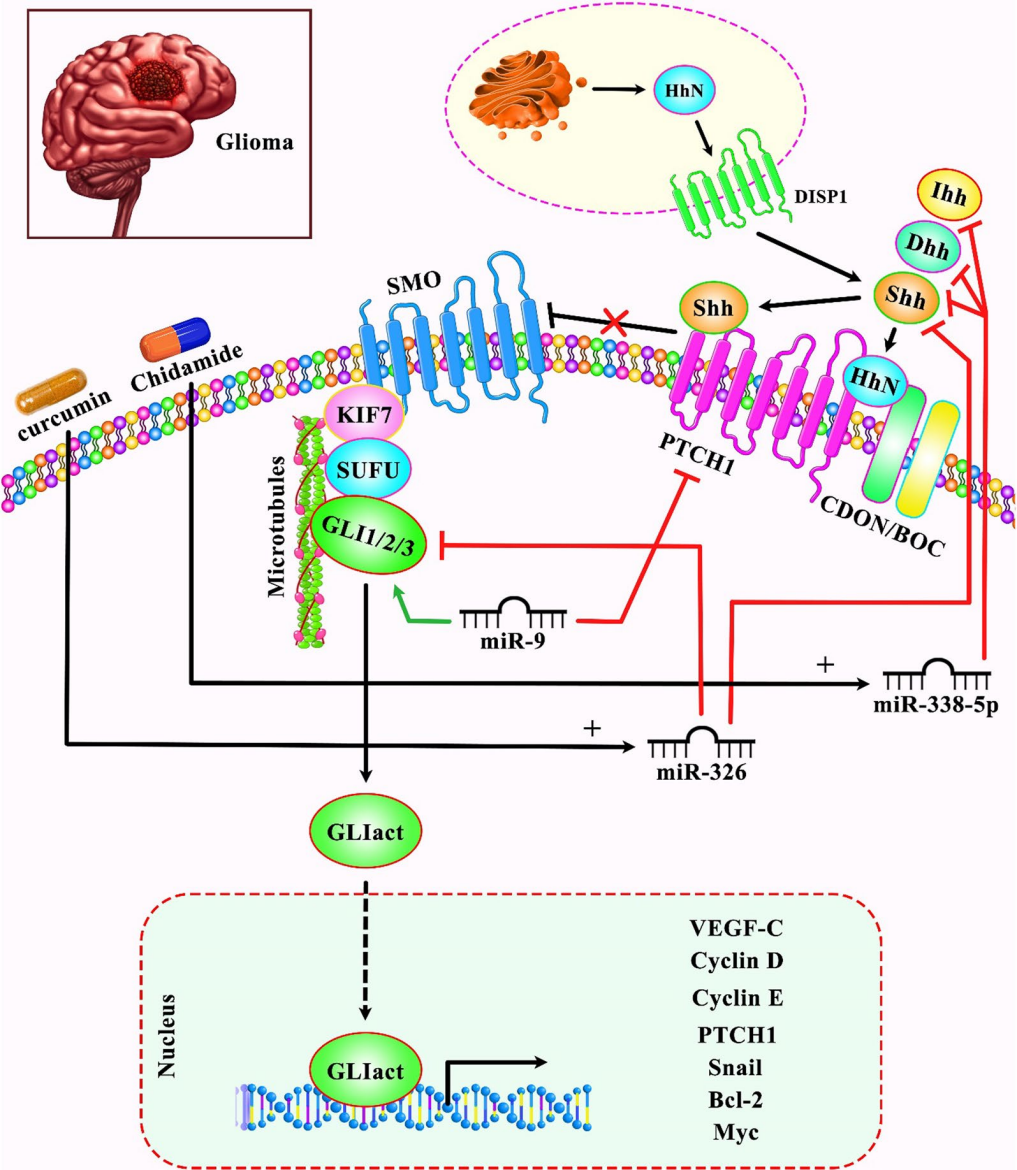
A schematic representation of the role of several miRNAs in regulating the sonic hedgehog signaling pathway in glioma. Accumulating evidence has revealed that upregulation of miR-326 in combination with curcumin can effectively contribute in the suppression of proliferation, and elevation of the apoptosis process in glioma cells via attenuating the activation of the SHH/GLI1 pathway [25]. Another finding confirms that miR-9 via targeting PTCH1 and promoting expression of GLI1 can trigger the activation of sonic hedgehog cascade and modulate expression of drug efflux transporters, MDR1 and ABCG2 in glioblastoma cells, therefore enhancing Temozolomide resistance in tumor cells [26]. Furthermore, mounting research has demonstrated that Chidamide can play an important role in inhibiting the expression levels of Shh, Ihh, and Dhh via upregulating miR-338-5p, thereby suppressing the growth rate, migration, and invasion of human malignant glioma cells. In fact, Chidamide exerts its effects by enhancing oxidative stress via the miR-338-5p-mediated regulation of Hedgehog pathway [27]

Shh-interacting ncRNAs can also affect the pathogenesis of brain tumors. For instance, miR-326 can effectively suppress proliferation, and induce apoptosis in glioma cells via attenuating the activation of the SHH/GLI1 pathway [25]. Moreover, miR-9 via targeting PTCH1 and enhancing expression of GLI1 can trigger the activation of Shh cascade and affect expression of drug efflux transporters, MDR1 and ABCG2 in glioblastoma cells, therefore enhancing Temozolomide resistance in tumor cells [26]. Figure 2 shows the role of Shh-interacting miRNA in glioma/glioblastoma.

Additional in vitro studies have revealed participation of Shh-related ncRNAs in several developmental processes as well as carcinogenic processes (Table 1).

**Table 1:** Interaction between ncRNAs and Shh signaling based on cell line studies

Tumor/Disease type or cellular mechanism	Targets/ regulators and signaling pathways	Cell line	Function	References
Alopecia	XIST, miR-424	3D-cultivated DP cells	↑↑ XIST: ↑ DP mediated hair follicle regeneration via targeting miR-424 to increase Shh expression	[16]
Breast cancer	miR-26a, FAM98A, SHH, SMO and GLI1	SK-BR-3, BT474, MDA-MB-231, MDA-MB-468, MCF-7, and MCF-10A	↑↑ miR-26a: ↓ proliferation, clone formation and metastasis, but ↑ sensitivity cells to docetaxel via targeting FAM98A, and reducing SHH, SMO and GLI1 expression levels	[20]
	LOC101930370, miR-1471, and Gli-1	MCF-7, MDA-MB-231, BT-474, SKBR3	∆ LOC101930370: ↓ cells progression via increasing miR-1471 and inhibiting SHH and Gli-1 expression	[21]
	lncRNA-Hh, Twist, Shh-GLI1 signaling, SOX and OCT4	MCF‐7, Hs578T, BT549, MDA‐MB‐231, human mammary epithelial cell line MCF10A	∆ lncRNA-Hh: ↓ the activity of Shh-GLI1 signaling	[22]
Breast cancer brain metastasis	circBCBM1, miR-125a/BRD4 axis, MMP9	231-BR cells	↑↑ circBCBM1: ↑ breast cancer brain metastasis via regulating miR-125a/BRD4 axis	[23]
Cardiopoesis	miR-210	embryonic stem cells	↑↑ SRF-dependent miR-210 expression: ↓ Shh signaling pathway activity via targeting of Shh, thus ↓ proliferation and cardiomyocyte progenitor differentiation	[17]
Cervical cancer	miR-129-5p, ZIC2	C-33A cell line and Hela	↑↑ miR-129-5p: ↓ invasion, migration and tumor angiogenesis via targeting ZIC2 and downregulating the Hedgehog signaling pathway	[28]
Congenital diaphragmatic hernia	miR-130a-5p, Foxa2	lung explant culture, HEK293T and BEAS-2B cells	∆ miR-130a-5p: ↓ CDH-associated abnormal branching morphogenesis ↑↑ miR-130a-5p: ↓ differentiation and ↑ apoptosis via targeting Foxa2 and in turn ↓ activation SHH signaling pathway	[18]
Craniofacial development	miR-199b, HIF1A, MAP3K4	chicken embryonic fibroblasts (DF-1 cells)	↑↑ miR-199b: ↓ SHH expression in the FEZ and craniofacial malformations via targeting HIF1A and MAP3K4	[19]
Diabetic foot ulcer	miR-155/ PTCH1 axis	EPCs	High glucose condition: ↑ miR-155 ↑↑ miR-155: ↑ impaired EPCs function by targeting PTCH1 (a receptor of shh signaling pathway)	[29]
Embryonal tumors	LIN28A, let-7, Wnt and Shh signaling	ETMRs	↑↑ LIN28A: ↓ maturation of let-7 microRNAs, thus modulating Wnt and Shh signaling let-7a-miRNA could target Gli1, Gli2, and Gli3 mRNAs Wnt and Shh signaling pathways are able to induce ETMR-like tumors	[30]
Embryonic cardiac malfunctions	miR-30c, Gli2 and Ptch1 (Shh signaling pathway)	P19 cells	↑↑ miR-30c ↑ proliferation by promoting cell entry into S phase but also ↓ apoptosis, and ↓ dimethyl sulfoxide-induced differentiation of P19 cells via targeting Gli2 and Ptch1, thus inhibiting the Shh signaling pathway	[31]
Embryonic lung development	miR-326, Arrestin β1	embryonic lung mesenchymal cells	Levels of miR-326 and its host gene, Arrestin β1, are increased in embryonic lung mesenchymal cells and Shh activity influences it miR-326: ↓ Shh signaling via directly targeting Smo and Gli2	[32]
Gallbladder carcinoma	SNHG6/miR-26b-5p axis	GBC-SD and NOZ	∆ SNHG6: ↑ cell apoptosis, ↓ growth, and ETM via upregulation of miR-26b-5p, thus inhibiting Gli1, Gli2, Shh, Smo, N-cadherin, vimentin and Snail, and promoting Gli3 and E-Cadherin expression	[33]
Glioblastoma Multiforme	miR-9, PTCH1, Gli 1	U87, T98G, HEK293, CCL64, BT145 and BT164	↑↑ miR-9: ↓ PTCH1 (via a SHH-independent method for TMZ resistance) and ↑ Gli 1 levels, thus activating the SHH pathway and also ↑ drug efflux transporters, MDR1 and ABCG2	[26]
	miR-326 and SHH/GLI1 pathway	U87 and U251	↑↑ miR-326: ↑ sensitivity of cells to curcumin via inhibiting SHH/GLI1 pathway	[25]
Glioblastoma	miR-137, RTVP-1, CXCR4, Shh and Nanog	U87, HF2354, HF2355 and HF2414	↑↑ miR-137: ↓ stemness of GSCs via targeting RTVP-1	[34]
Glioma	miR-338-5p	U87 and HS683 glioma cells	Chidamide: ↓ glioma cells via increasing oxidative stress by the miR-338-5p regulation of Hedgehog signaling	[27]
Hepatocellular carcinoma	TUG1/ miR-132/shh axis	LM3, HepG2, Hep3B, Huh7, SMMC7721 and MHC97H, and LO2	↑↑ miR-132: ↓ proliferation but ↑ apoptosis via targeting shh ∆ TUG1: ↓ proliferation via targeting miR-132 and increasing Shh protein expression	[35]
Inflammatory bowel disease	NOD2, miR-146a, and NUMB	macrophages	NOD2-induced miR-146a activates SHH signaling via targeting NUMB NOD2-iNOS/NO-miR-146a-mediated SHH Signaling increases expression of inflammatory genes	[36]
Laryngeal cancer	LINC‐PINT/ miR‐425‐5p/PTCH1 axis	HEp‐2	↑↑ LINC‐PINT: ↓ cisplatin resistance and stemness via targeting miR‐425‐5p and upregulating PTCH1 (a protein of the Hedgehog pathway)	[37]
Liver fibrosis	miR-200a/Gli2 axis	HSCs from male Sprague–Dawley rats	↑↑ miR-200a: ↓ proliferation and ↓ EMT via targeting Gli2	[38]
Lung cancer	miR-506/SHH axis	HCC4006	↑↑ miR-506-3p: ↑ sensitivity EGFR-TKI-resistant cells to Erlotinib-induced cell death, ↑ E-cadherin expression, bur ↓ SHH signaling, ↓ vimentin, thus ↓ the EMT-mediated chemoresistance	[39]
	BLACAT1	A549 and PC9 cells	↑↑ BLACAT1: ↑ proliferation, migration and invasion via activating shh signaling pathway, by inducing shh, Gli-1 and Smo expression	[40]
	LINC01426, USP22	HBE, H1299, A549, PC-9, Calu3	∆ LINC01426: ↓ proliferation, migration, EMT, and stemness ↑↑ LINC01426: ↑ LUAD progression via recruiting USP22 to stabilize SHH protein	[41]
Lung fibrosis	miR-193a, PI3K/Akt/mTOR and hedgehog signaling	A549 cells	↑↑ miR-193a: ↑ p-Akt, Beclin1 and LC3-II levels, thus ↑ autophagy	[42]
M. bovis BCG infection of DCs	COX-2, PD-L1, miR-324-5p and miR-338-5p	Dendritic cells	SHH signaling is required for Treg expansion in DCs via increasing COX-2 and PD-L1 and inhibiting miR-324-5p and miR-338-5p PD-L1 was a direct target of miR-324-5p and miR-338-5p	[43]
Medulloblastoma	Arrb1, miR-326, Hh/Gli pathway	CSCs	↑↑ miR-326: ↓ proliferation and self-renewal by decreasing Smo and Gli2	[44]
	Shh/Gli2/Nkx2-2as axis	Daoy and D341 Med, and HEK293T	Shh/Gli2 reduces Nkx2-2as levels by transcriptionally activating FoxD1 ↑↑ Nkx2-2as1: ↓ tumorigenesis	[45]
Myocardial ischemic/reperfusion (I/R) injury	SNHG6/miR-135a-5p/HIF1AN axis and Shh/Gli1 signaling	H/R-induced cardiomyocytes	↑↑ SNHG6: ↑ H/R-induced apoptosis in cardiomyocytes via regulating miR-135a-5p/HIF1AN axis and inactivating Shh/Gli1 signaling	[46]
Neuroblastoma	miR181-a and –b, CDON	MYCN, SH-SY-5Y, SK-N-AS, IGR-N-91, NiH-3T3	↑↑ CDON: ↑ apoptosis CDON (a receptor for SHH) is regulated by miR181-a and –b	[47]
Osteoarthritis	miR-602, miR-608	Chondrocytes and HEK 293 cells	↑↑ miR-602 and miR-608: ↓ activity of pMIR-REPORT-Luc-SHH reporter vector and ↓ IL-1β-induced endogenous SHH mRNA and protein expression in OA chondrocytes	[48]
Osteogenesis	miR-342-3p/Sufu axis, TGF-β signaling pathway	HUCMSCs	↑↑ miRNA-342-3p: ↑ expression of osteogenic genes, ↑ osteogenic differentiation of hUCMSCs and ↑ TGF-β signaling pathway via targeting Sufu	[49]
Osteogenic differentiation	miR-342-3p/Sufu axis	UCMSCs	↑↑ miR-342-3p: ↑ osteogenic differentiation via targeting Sufu and activating Shh signaling pathway	[50]
Osteonecrosis of the femoral head	miR-378-ASCs-Exos, Sufu	BMSCs and HUVECs	↑↑ miR-378-ASCs-Exos: ↑ osteogenesis and angiogenesis via targeting Sufu to increase the Shh signaling pathway, thus reducing GC-induced ONFH development	[51]
Pancreatic cancer	miR-132	MiaPaCe-2a	↑↑ miR-132: ↑ proliferation and apoptosis via targeting Shh	[24]
Retina injury	Shh Signaling/lin28a/let-7 axis	BrdU + and BrdU − and HEK293T	Downregulation of let-7 by lin28a is necessary for the regulation of Shh signaling as a part of positive feedback loop induced via the Ascl1a-lin28a axis, in turn is essential for retina regeneration	[52]
Retinoblastoma	miR-361-3p/ GLI1/3 (shh signaling) axis	Y79 and Weri-Rb-1	↑↑ miR-361-3p: ↓ proliferation and stemness via targeting GLI1 and GLI3	[53]
Skin wound healing	VEGF, miR-200 family, E-cadherin	mouse ESCs	↑↑ shh: ↑ migration and skin wound healing via increasing VEGF and negatively regulating the transcription of the miR-200 family, thus downregulating E-cadherin	[54]
Stroke	miR17-92 cluster	SVZ neural progenitor cells from adult mice	↑↑ miR17-92 Cluster: ↑ proliferation and survival of SVZ neural progenitor cells miR17-92 cluster expression is mediated by Shh signaling	[55]
Thyroid cancer	miR-141-3p, BRD4, and PI3K/AKT pathways	Nthy-ori 3–1 and TPC-1	Propofol treatment: ↓ proliferation, migration, and invasion via ↑ miR-141-3p, and in turn ↓ BRD4, thus inhibiting the activity of SHH and PI3K/AKT pathways	[56]

*MGPC* Müller glia-derived progenitor cells, *DCs* dendritic cells, *OA* Osteoarthritis, *EMT* epithelial-mesenchymal transition, ∆ knock-down, deletion, *TCRV* triacetyl resveratrol, *TMZ* temozolomide, *MFE* mammosphere-formation efficiency, *LUAD* lung adenocarcinoma

## Animal studies

Animal studies have shown participation of Shh-related ncRNAs in a variety of non-neoplastic disorders, namely acute myocardial infarction, alopecia, cerebrovascular disorders, diabetes mellitus, inflammatory bowel disease, lung fibrosis, osteoporosis, Parkinson's disease and trigeminal neuralgia as well as different types of cancers (Table 2). For instance, knock-down of miR-802-5p has resulted in reduction of cell apoptosis after myocardial infarction through enhancing activity of Shh signaling, thus decreasing myocardial injury and improving cardiac function [57]. Moreover, experiments in animal models have shown up-regulation of XIST increases dermal papilla cells-mediated hair follicle regeneration via targeting miR-424 to promote Shh expression, thus activating hedgehog signaling [16]. Moreover, miR-153 has been found to decrease expression of PTC expression and enhance activity of Shh signaling pathway to increase angiogenesis in a rat model of cerebral ischemic injury [58].

**Table 2:** Interaction between ncRNAs and Shh pathway based on studies in animal models

Tumor/disease type or cellular mechanisms	Animal models	Results	References
Acute myocardial infarction	6–8-week-old male Sprague Dawley (SD) rats (Rat MI Model)	∆ miR-802-5p: ↓ apoptosis after MI via activating Shh signaling pathway via targeting PTCH1, thus decreasing myocardial injury and improving heart function	[57]
Alopecia	DP sphere xenograft to nude mice	↑↑ XIST: ↑ DP mediated hair follicle regeneration via targeting miR-424 to promote Shh expression, thus activating hedgehog signaling	[16]
Breast cancer	5 weeks‐old athymic nude mice	∆ lncRNA‐Hh: ↓ tumor growth	[22]
Breast cancer brain metastasis	6-week-old female BALB/c nu/nu mice	∆ circBCBM1: ↓ tumor volumes and weights	[23]
Cerebrovascular disease	rat cerebral ischemic injury model	miR-153 expression was decreases ↑↑ miR-153: ↓ PTC expression and ↑ activation of Shh signaling pathway and angiogenesis	[58]
Cervical cancer	4-week-old SPF female BALB/c nude mice	↑↑ microRNA: ↓ tumor growth and tumor angiogenesis via targeting ZIC2 and downregulating the Hedgehog signaling pathway	[28]
Diabetes mellitus	Sprague–Dawley male rats	miR-9 and miR-29a: ↓ activation of SHH signaling pathway via ISL1, nociception threshold and peripheral nerve conduction velocity miR-9 and miR-29a rise AR activity and disease activity by reducing ISL1	[59]
Glioblastoma multiforme	5-week–old female BALB/c-nude mice	↑↑ miR-326 combined with curcumin treatment: ↓ tumor growth	[25]
Hepatocellular carcinoma	4-week-old male athymic BALB/c nu/nu mice	↑↑ TUG1: ↓ tumor growth via targeting miR-132	[35]
Inflammatory bowel disease	C57BL/6 wild-type (WT) and iNOS − / − mice	NOD2-iNOS/NO-miR-146a-mediated SHH Signaling is necessary for inflammatory responses	[36]
Liver regeneration	carbon tetrachloride (CCl4)-treated rats transplanted with human CP-MSCs (Tx) or saline (non-Tx)	↑↑ miR-125b from CP-MSCs: ↓ activation of Hh signaling, thus ↑ the reduced fibrosis	[60]
Lung cancer	6-week-old female BALB/C nude mice	∆ BLACAT1: ↓ tumor growth and metastasis	[40]
	4-week-old BALB/c nude female mice	∆ LINC01426: ↓ tumor size, volume, and weight	[41]
Lung fibrosis	6-week-old female C57BL/6 mice	↑↑ miR-193a: ↑ autophagy and ↓ PQ-induced pulmonary fibrosis Ligustrazin: ↑ autophagy and ↓ paraquat-induced pulmonary fibrosis	[42]
Medulloblastoma	Ptch1 ± mice, C57BL/6 and PtenFloxp/Floxp mice, GFAP-Cre mice	The effects of miR-183∼96∼182 to maintain cell proliferation depends on hedgehog signaling activation	[61]
	Athymic nude mice	↑↑ Nkx2-2as: ↓ tumor growth Gli2/FoxD1/Nkx2-2as axis was found to be involved in the pathogenesis of Shh-subtype MB	[45]
Neuroblastoma	17-day-old chick embryos	↑↑ CDON: ↓ tumor size	[47]
Osteonecrosis of the femoral head	a rat model of GC-induced ONFH	↑↑ miR-378-ASCs-Exos: ↑ osteogenesis via targeting Sufu to increase the Shh signaling pathway	[51]
Osteoporosis	Wistar female rats	Levels of SUFU were upregulated bet levels of miR-874, Shh, Ptch, Smo, BMP2, Runx2, and PCNA were downregulated ↑↑ miR-874: ↑ proliferation and differentiation of via targeting SUFU and activating of Hedgehog signaling pathway	[62]
Parkinson's disease	male specific pathogen-free C57BL/6 mice	↑↑ miR-124: ↑ proliferation and ↓ apoptosis by downregulating EDN2 through activating the Hedgehog signaling pathway	[63]
The transition of dividing neuroepithelial progenitors to differentiated neurons and glia	zebrafish	∆ miR-219: ↑ growth of primary cilia via elevating Shh signaling	[64]
Trigeminal neuralgia	rat model of CCI-IoN	Upregulation of miR-195 and downregulation of Patched1 were seen ↑↑ miR-195: ↑ facial pain development via targeting Patched1 in the Shh signaling pathway	[65]

*∆* knock-down, deletion, *AR* aldose reductase, *MI* myocardial infarction, *SPF* specific pathogen free, *MB* Medulloblastoma

Experiments in animal models of breast cancer have verified that knock-down of lncRNA-Hh [22] and circBCBM1 [23] can led to reduction of tumor growth. Meanwhile, miR-326 has been shown to increase effects of curcumin in animal models of glioblastoma through modulation of Shh/GLI1 signaling pathway [25].

## Studies in clinical samples

Expression assays in clinical samples from a variety of tumor types have indicated down-regulation of Shh-targeting miRNAs such as miR-26a [20] and miR-1471 [21] in breast cancer, miR-129-5p in cervical cancer [28], miR-361-3p in retinoblastoma, miR-26b-5p in gallbladder carcinoma [33], and miR-361-3p in retinoblastoma [53]. In gallbladder cancer, dysregulation of miR-26b-5p has been associated with age and sex of patients, tumor invasion, differentiation degree, tumor location, and TNM stage [33]. Conversely, an expression assay in pancreatic cancer samples has shown up-regulation of miR-132 and down-regulation of Shh [24]. In neuroblastoma samples, down-regulation of CDON and up-regulation of miR-181-a and miR-181-b have been associated with poor overall survival, higher tumor stage and more aggressive phenotype [64].

Shh-related lncRNAs are also involved in the process of keloid formation. Expression assays in keloid tissues and adjacent normal skin epidermis have shown differential expression of 30 lncRNAs and 33 mRNAs between these two sets of samples. Dysregulated lncRNAs included up-regulated lncRNAs AK055628, MIAT, MIR31HG, RP11-264F23.3, and AC073257.2, and downregulated lncRNAs RP11-12M9.3, XLOC_007437, XLOC_009485, RP5-1042I8.7, and HNF1A-AS1 [66].

Table 3 summarizes dysregulation of Shh signaling-related ncRNAs and in clinical samples.

**Table 3:** Dysregulation of Shh signaling-related ncRNAs and in clinical samples

Tumor/Disease type or different Cellular Mechanisms	samples	Expression (Tumor vs. Normal)	Kaplan–Meier analysis (impact of Shh regulators dysregulation)	Association of dysregulation of Shh regulators with clinical characteristics	References
Breast cancer	13 pairs of tumor/nearby tissues	Down-regulation of miR-26a	–	–	[20]
	15 pairs of tumor/nearby tissues	Down-regulation of miR-1471	–	–	[21]
Cervical cancer	87 pairs of tumor/nearby tissues	Down-regulation of miR-129-5p, and activated Hedgehog signaling pathway	–	–	[28]
Diabetes mellitus	GEO database (GSE27382 and GSE95849) 30 patients with DM	Up-regulation of miR-9 and miR-29a Down-regulation of ISL1	–	–	[59]
Gallbladder carcinoma	68 gallbladder cancer patients and 70 healthy controls	Up-regulation of SNHG6 and down of miR-26b-5p	–	age, sex, tumor invasion, differentiation degree, tumor location, and TNM staging	[33]
Glioblastoma Multiforme	TCGA dataset with > 500 different GBM samples	Up-regulation of miR-9	–	–	[26]
Hepatocellular carcinoma	20 pairs of tumor/nearby tissues	Up-regulation of SHH and down of miR-132	–	–	[35]
Laryngeal cancer	24 pairs of tumor/nearby tissues	Down-regulation of LINC‐PINT and up of miR-425-5p	–	stemness	[37]
Lung cancer	20 pairs of tumor/neraby tissues	Up-regulation of BLACAT1	–	–	[40]
	GEPIA database: 483 tumor tissues and 347 normal tissues	Up-regulation of LINC01426	–	–	[41]
Neuroblastoma	226 NB patients	Down-regulation of CDON and up-regulation of miR-181-a and miR-181-b	Poor OS	higher-staged, more aggressive tumors	[64]
Osteoarthritis	46 OA patients	Up-regulation of SHH and it signaling targets	–	–	[48]
Pancreatic cancer	23 pancreatic adenocarcinomas, 18 adjacent benign pancreatic specimens and 25 normal pancreatic specimens	Up-regulation of miR-132 and down-regulation of Shh	–	–	
Retinoblastoma	10 patients with RB and 10 healthy controls	Down-regulation of miR-361-3p	–	–	[53]

*GEO* Gene Expression Omnibus, *DM* Diabetes mellitus, *OA* osteoarthritis, *GBM* Glioblastoma Multiforme, *TNM* tumor-node-metastasis

## Discussion

Shh signaling is involved in a variety of cellular functions, including tissue development and regeneration, stem cell functions and carcinogenesis. Thus, it is not surprising that ncRNAs that regulate activity of this pathway are implicated in the pathogenesis of a wide range of human disorders. In fact, this pathway represents a prototype of shared pathways between embryogenesis and carcinogenesis.

In the context of malignant disorders, Shh-interacting ncRNAs not only affect cancer progression, but also determine response of cancer cells to a variety of anticancer therapies. Both functions can be explained by the crucial roles of this pathway in the induction of stemness. However, at least in some types of cancers, Shh signaling seems to have protective effects against carcinogenesis. For instance, in pancreatic cancer, expression of the Shh-targeting miRNA miR-132 has been found to be up-regulated parallel with down-regulation of Shh levels [24]. Shh pathway can also induce epithelial-to-mesenchymal transition in gastric, pancreatic, and bladder cancers [67–69]. Thus, Shh-interacting ncRNAs might also affect this feature.

It is estimated that one-third of malignancies are correlated with abnormal activity of the Hh signaling pathway [70]. Dysregulation of Hh signaling can contribute to the imitation, growth, metastasis, and apoptosis of several types of cancers. In fact, three patterns of induction of the Hh signaling cascade have been identified in several cancers. These patterns are ligand independent oncogenic Hh pathway, autocrine or juxtacrine and paracrine or reverse paracrine patterns [70].

Moreover, there are several examples of interactions between two classes of ncRNAs in the context of regulation of activity of Shh signals. XIST/miR-424, LOC101930370/miR-1471, circBCBM1/miR-125a, TUG1/miR-132, SNHG6/miR-26b-5p, LINC‐PINT/miR‐425‐5p, and SNHG6/miR-135a-5p are examples of lncRNA/miRNA or circRNA/miRNA pairs that cooperatively regulate activity of Shh pathway. The regulatory impact of these axes on function of Shh pathway should be assessed in different cellular and disease contexts to find whether they act in a context-specific manner or a ubiquitous manner. This has importance in design of novel therapies for each disorder in which abnormal function of Shh pathway has been detected. However, circRNAs have limited roles as miRNA sponges in most cases [71–74]. In fact, most circRNAs are much less abundant than miRNAs and are not predicted to function as miRNA sponges [75, 76].

Although Shh-related ncRNAs are expected to influence the prognosis and clinical outcome of cancer, this issue has been verified only in the gallbladder cancer, neuroblastoma and laryngeal cancer. Thus, future studies should assess the prognostic roles of these ncRNAs as well as their potential as diagnostic markers.

A number of anti-cancer agents such as propofol have been found to exert their effects through modulation of Shh-related ncRNAs. This agent has been found to simultaneously decrease activity of Shh and PI3K/AKT pathways [48]. Therefore, modulation of expression of Shh-related ncRNAs is a promising anticancer strategy.

Taken together, the data presented above indicates contribution of several ncRNAs in the regulation of Shh pathway and their involvement in the pathogenesis of several disorders. However, this data has some limitations. No comprehensive assessments of different types of ncRNAs using next generation sequencing techniques have been performed. Thus, the interactive networks between different types of ncRNAs and Shh signaling components have not been identified yet.

A number of Hh inhibitors, namely Smo antagonist, Cyclopamine, Sulforaphane, Baicalein, Sangunarine, GANT61, Sonidegib, and PF-04449913 have been used for inhibition of cancer stem cells [77]. Meanwhile, activity of cancer stem cells has been shown to be affected by a number of mentioned ncRNAs. Therefore, combination of mentioned therapeutic modalities with ncRNA-targeted therapies might be regarded as effective options for eradication of cancer stem cells.

Since Shh-related ncRNAs have fundamental roles in the pathogenesis of human disorders, it is possible to down-regulate or up-regulate their expression in order to alter the pathological events in the course of disease evolution. In order to translate the basic science about the role of ncRNAs in the regulation of Shh pathway into clinical application, the following steps should be followed: (1) comprehensive assessment of expression of different classes of ncRNAs in clinical samples; (2) application of system biology methods for analysis of the acquired data; (3) understanding the complex network between different classes of ncRNAs and components of Shh pathway; (4) establishment of in vitro and in vivo models for assessment of the function of each module and (5) finding novel modalities for influencing the expression and activity of these modules.

## Data Availability

The analyzed data sets generated during the study are available from the corresponding author on reasonable request.

## Abbreviations

Shh: Sonic Hedgehog
Gli: Glioma-associated oncogene
Hh: Hedgehog
cAMP: Cyclic adenosine 3', 5'-monophosphate
Dhh: Desert hedgehog
Ihh: Indian hedgehog
SRF: Serum response factor
lncRNAs: Long non-coding RNAs
miRNAs: MicroRNAs
circRNAs: Circular RNAs
GEO: Gene Expression Omnibus
DM: Diabetes mellitus
OA: Osteoarthritis
GBM: Glioblastoma Multiforme
TNM: Tumor-node-metastasis
